# Prevalence of metallo-β-lactamase producing *Pseudomonas aeruginosa* and *Acinetobacter* species in intensive care areas in a tertiary care hospital

**DOI:** 10.4103/0972-5229.76089

**Published:** 2010

**Authors:** Anuradha S. De, Simit H. Kumar, Sujata M. Baveja

**Affiliations:** **From:** Department of Microbiology, L.T.M. Medical College, Mumbai, India

**Keywords:** MBL producers, ICUs

## Abstract

A total of 39 non-duplicate isolates of carbapenem-resistant *Pseudomonas aeruginosa* and *Acinetobacter* species isolated from blood and endotracheal secretions were tested for metallo-β-lactamase (MBL) production by modified-EDTA disc synergy and double disc synergy tests. The prevalence of MBLs was 33.33% by both the above tests. All patients with MBL-positive isolates were multidrug resistant and had multiple risk factors like > 8 days hospital stay, catheterization, IV lines, previous antibiotic use, etc. These were risk factors for imipenem resistance also. The overall mortality in MBL-positive patients was 46.15%.

## Introduction

Metallo-β-lactamases (MBLs) are metalloenzymes of Ambler class B and are clavulanic acid-resistant enzymes. They require divalent cations of zinc as co-factors for enzymatic activity and are universally inhibited by ethylenediamine tetra-acetic acid (EDTA), as well as other chelating agents of divalent cations.[1] The first plasmid-mediated MBL was reported in *Pseudomonas aeruginosa* in Japan in 1991.[2] Since then many countries including few reports from India are available regarding the prevalence of MBLs.[3–8] The present study was conducted to determine the prevalence of MBLs in *P. aeruginosa* and *Acinetobacter* species in intensive care areas.

## Materials and Methods

A total of 39 imipenem resistant, non-duplicate isolates of *P. aeruginosa* (14) and *Acinetobacter* species (25) were recovered from blood and endotracheal secretions of patients from intensive care areas of Lokmanya Tilak Municipal Medical College and Hospital during 1.5 year period (April 2007 to September 2008). Imipenem resistance was determined by the Kirby-Bauer disc diffusion method (KBDDM) and then were further tested for MBL production by the modified-EDTA disc synergy test (MDST)[9] and the double disc synergy test (DDST).[10] Antibiotic susceptibility of all MBL isolates was performed on Mueller Hinton agar by KBDDM according to CLSI guidelines.[11] Antibiotics tested were gentamicin, amikacin, netilmycin, amoxicillin-clavulanic acid, cefotaxime, ceftriaxone, ceftazidime, cefepime, ciprofloxacin, ofloxacin, piperacillin and piperacillin-tazobactam. A proforma was prepared and filled up for each patient, from whom MBL producing organisms were isolated.

## Results

Out of 39 imipenem-resistant isolates, 24 (61.54%) were from adults from Medical Intensive Care Unit (MICU) and 15 (38.46%) from children from Paediatric Intensive Care Unit (PICU). Of 39, twenty one were from endotracheal secretions and 18 from blood. The overall prevalence of MBLs was 33.33% (13/39) in this study, by both MDST and DDST, of which 28.57% (4/14) were *P. aeruginosa* and 36 % (9/25) were *Acinetobacter* species.

All MBL-positive isolates were resistant to all the antibiotics tested. A majority of MBL-positive isolates were from males (58.97%). Out of 13 MBLs, 10 (76.92%) were from MICU and 3 (23.08%) from PICU.

Table 1 shows the risk factors in patients with MBL-positive and -negative isolates of *P. aeruginosa* and *Acinetobacter* species. All 39 imipenem-resistant isolates had the first four risk factors [Table 1]. The mortality in patients with MBL-positive isolates was 46.15% (6/13) and with MBL-negative isolates was 11.54% (3/26). Out of six deaths due to MBL, 50% (2/4) were due to *P. aeruginosa* and 44.44% (4/9) due to *Acinetobacter* species.

**Table 1:** Risk factors in patients with MBL-positive and -negative isolates of *Pseudomonas aeruginosa* and *Acinetobacter* species

Risk factors	Pseudomonas aeruginosa (14)		Acinetobacter species (25)		Total (39)	
	MBL + (04)	MBL − (10)	MBL + (09)	MBL − (16)	MBL + (13) No. (%)	MBL − (26) No. (%)
Duration of hospital stay >8 days	04	10	09	16	13 (100)	26 (100)
Catheterization	04	10	09	16	13 (100)	26 (100)
Intravenous line	04	10	09	16	13 (100)	26 (100)
Previous antibiotic use	04	10	09	16	13 (100)	26 (100)
Mechanical ventilation	03	08	09	15	12 (92.31)	23 (88.46)
Endotracheal intubation	03	08	07	14	10 (76.92)	22 (84.62)
Fetal risk factors* (15)	00	02/03	02/03	06/09	2/3 (66.67)	8/12 (66.67)
Maternal risk factors** (15)	00	02/03	02/03	06/09	2/3 (66.67)	8/12 (66.67)

* Low birth weight and prematurity in all

** Premature rupture of membrane in four, pregnancy induced hypertension in five and anemia in one.

## Discussion

As MBLs will hydrolyze virtually all classes of β-lactamase, there continued spread will be a clinical catastrophe.[1] With the global increase in the types of MBLs, early detection is crucial.[5] Over the last decade, most of the studies were on different methods of MBL detection in *P. aeruginosa* and *Acinetobacter* species.[3,4,5,6] Though MIC detection is gold standard, DDST and MDST are comparable with the former and at the same time are simple, reliable, less cumbersome and cheap, as per previous reports.[3–5] Lee *et al*, have reported 100% sensitivity and specificity of MDST.[9] Therefore, these tests can be used in a small laboratory set up also. Using both these tests, the prevalence of MBL was found to be 33.33% in this study. The prevalence of MBLs in *P. aeruginosa* was lower (28.57%) than in *Acinetobacter* species (36%). Our prevalence of MBLs in *Pseudomonas* correlates well with other studies (30.3% - 36%)[3–5]. One Indian study has reported very high prevalence (80%).[7] Yong *et al*,[12] have reported 26.5% MBLs in *Acinetobacter* species.

Apart from being imipenem resistant, MBLs were resistant to important groups of antibiotics tested, including the third-generation cephalosporins, aminoglycosides and quinolones – a characteristic feature of MBL producers.[1,3] For MBLs, limited treatment options are available and the only therapeutic option may be polymyxins, but it should not be used as monotherapy.[1] It can be combined with an appropriate aminoglycoside. Aztreonam is the drug of choice for MBL producing *Pseudomonas aeruginosa*.[1] Combination therapy is often employed in treatment of multidrug-resistant *Acinetobacter baumanii*. Imipenem or meropenem combined with ampicillin-sulbactam is active against carbapenem-resistant as well as MBL-positive strains of *Acinetobacter* species.[13]

Multiple risk factors (four or more) were present in all patients with MBL-positive isolates. All had risk factors of hospital stay > 8 days, catheterization, IV line and previous antibiotic use [Table 1]. Interventions like mechanical ventilation and endotracheal intubation were in 92.31% and 76.92% MBL-positive patients, respectively. All the above were major risk factors for imipenem resistance also. We undertook this study to find out the risk factors for MBL acquisition. We concluded that there is no separate risk factor for MBL acquisition, as compared to MBL-negative, imipenem-resistant isolates. Risk factors for both the above were same in this study. Infection Control Fact Sheet of 2007 of a hospital mentions possible risk factors for acquisition of MBLs as prolonged hospitalization; prior antimicrobial therapy; treatment in ICU and haematology, where antibiotic usage is high.[14] In this study also, we reported 13 MBLs from intensive care areas (ICUs) and all had hospital stay > 8 days and previous antibiotic use [Table 1].

The mortality of MBL-positive patients was 46.15% in this study. A recent study has reported the same in 57% patients.[7] Patients with MBL producing Pseudomonas had a higher mortality (50%) than *Acinetobacter* species (44.44%), in accordance with other studies.[3,4] One study has reported more mortality due to *Acinetobacter baumanii* (68%) than with *P. aeruginosa* (47%).[7]

Emergence of MBL producing *P.aeruginosa* and *Acinetobacter* species in ICUs is alarming and reflects excessive use of carbapenems. Intensity of selection pressure for usage of broad spectrum antibiotics is high in ICUs, resulting in eradication of competitive flora and selection of multidrug-resistant strains.[7] Therefore a strict antibiotic policy should be followed in intensive care areas to prevent further spread of MBLs. Clinicians should prescribe antibiotics judiciously. Timely implementation of strict infection control practices and antibiotic resistance surveillance programs should be carried out from time to time.[5] Detection of MBLs by either DDST or MDST should be routinely performed in all microbiology laboratories for all imipenem-resistant isolates, which will help to reduce morbidity and mortality in these patients.
